# Stokes-Adams' Disease

**Published:** 1905-12-09

**Authors:** 


### STOKES-ADAMS' DISEASE.

The curious collection of symptoms which goes by the name of Stokes-Adams' disease is uncommon but not extraordinarily rare. Dr. Gossage[1] has found records of some hundred cases in the literature. The distinctive features of the malady are: (*a*) An extremely infrequent pulse, which is usually associated with a similar infrequency of the heart's action, although occasionally intermediate beats may be heard over the heart which are too weak to produce a pulsation at the wrist. (*b*) Certain nervous symptoms. These may be divided into three groups: (1) Syncopal attacks, in which the patient falls down suddenly with complete loss of consciousness, shallow breathing, and feeble pulse, the seizure lasting a few minutes. These severe attacks may be preceded by, or may alternate with, attacks of slight vertigo. (2) Epileptiform attacks, in which, in addition to the unconsciousness, there is some degree of convulsion. (3) Apoplectiform attacks, throwing the sufferer suddenly into a deep coma accompanied by stertorous breathing and cyanosis, but resulting in no subsequent paralysis.

The cause of these attacks is obscure. The author states that the majority of cases occur in males during the later degenerative period of life in association with considerable arterial sclerosis. Stokes, in his original description, considered the phenomena to depend upon fatty degeneration of the heart-muscle, and Dr. Osler considers that when the disease sets in after an acute specific fever, the probable cause is an acute degenerative myocarditis. Huchard speaks of this syndrome as "the cardio-bulbar form of arteriosclerosis," and holds the symptoms to be an expression of sclerosis in the arteries to the spinal bulb. Dr. Gossage points out that such an explanation would exclude the instances where the disease has attacked young subjects. In some of these last there have been found valvular lesions of the heart, in others gross lesions of the nervous system, while in the remainder there has been no discoverable lesion either of the vascular or of the nervous apparatus.

As a rule the infrequency of the pulse is permanent, though occasionally this peculiarity is only to be observed at the time of the nervous attack. In the majority of cases, also, the pulse, though infrequent at all times, becomes less frequent than ever during the attack. Osler has reported the case of a victim of this disorder over whose heart, on the occasion of an epileptiform attack of the kind described above, no sound whatever was heard for a space of thirty-five seconds. Such patients react little or not at all to the ordinary causes of cardiac acceleration. Exertion and emotion affect the pulse-rate hardly at all, though they may actually further reduce the already infrequent pulsations, and drugs like atropine and digitalis are said to be inactive as regards the pulse-rate of these patients.

A patient reported by Huchard passed through an attack of pneumonia, in the course of which, though the body-temperature rose to 104° F., the pulse-rate was only accelerated to the extent of eight beats a minute, that is to say, from 36 to 44. The subjects of the disease invariably complain of coldness of the extremities.

The prognosis is very grave, for death may occur suddenly during one of the nervous attacks. Those who have an associated arteriosclerosis or renal disease, are likely to exhibit the symptoms peculiar to these maladies, such, for instance, as Cheyne-Stokes' breathing, dyspnœa of an asthmatic type, or angina pectoris. In rare instances the duration of the disease may be very prolonged. Osler has recorded the case of a man who, after enduring a thirty years' infliction of slow pulsation accompanied by fainting attacks, died in one of the attacks at the age of seventy-four. Where the disease follows acute fevers complete recovery may ensue, and even in the presence of arteriosclerosis amelioration of the symptoms may occasionally be observed.

Of the three cases detailed the first was that of a woman aged fifty-eight years, who for a month before she came under observation had been subject to attacks of giddiness coming on three or four times a week. The patient was intellectually too dull to give a clear account of her experiences, but an eye-witness of the seizures stated that they would attack the patient quite suddenly, she would fall to the ground apparently unconscious, " become a sort of dead colour, and go all of a twitch." The seizures usually lasted from five to ten minutes.

This patient showed many lesions of tertiary syphilis, her pulse-rate was 40, her radial artery hard, and the physical examination of the heart negative except for slowness of pulsation.

Case 2 was that of a young man of twenty-three years, who suffered for about four years from attacks of fainting, in one of which he died. His pulse-rate was 36, and his heart was hypertrophied considerably. There was a systolic bruit over the aortic area but no thrill. Dr. Gossage does not state whether the case should be considered an example of the rare valvular lesion, pure aortic stenosis.

Case 3 was that of a man aged sixty-nine, who was suddenly attacked by unconsciousness in the street. Since his first seizure he had suffered several every day, but minor ones as a rule, though occasionally sufficient to cause his fall. He was an anæmic man, with marked albuminuria, atheromatous arteries, an increased pulse-tension, and a pulse-rate of 32. On one occasion he had an attack of unconsciousness lasting ten minutes, in which there was distinct twitching of the muscles of the face and upper limbs. Five days later his pulse-rate had risen to 84, but whether the higher rate was maintained or not is, unfortunately, not stated.

[1] Clin. Soc. Trans., 1905.

